# Fracture resistance of bonded ceramic overlay restorations prepared in various designs

**DOI:** 10.1038/s41598-022-21167-7

**Published:** 2022-10-05

**Authors:** Wilasinee Channarong, Nuttakarn Lohawiboonkij, Pitsinee Jaleyasuthumkul, Kittipong Ketpan, Nut Duangrattanaprathip, Kornchanok Wayakanon

**Affiliations:** 1 Department of Oral Biology, Naresuan University, Phitsanulok, Thailand; 2 Department of Restorative Dentistry, Naresuan University, Phitsanulok, Thailand

**Keywords:** Health care, Materials science

## Abstract

This study investigates fracture resistance of adhesive ceramic overlays of various designs. Forty-eight upper premolar teeth were divided into eight groups. The variations were: shoulder margins on the buccal and lingual surfaces with axial wall heights of 1, 2, or 3 mm; one shoulder margin with axial wall height of 1, 2, or 3 mm on the lingual surface and one contrabevel margin on the buccal surface; contrabevel margins on the buccal and lingual surfaces; and a control of sound teeth. Overlays were designed and fabricated with CAD/CAM using zirconia-reinforced lithium disilicate ceramic and bonded with resin cement. Samples underwent thermocycling and dynamic fatigue equivalent to 6 months of use. Compressive loading was applied until fracture, and fracture mode was analyzed. Results showed no statistical difference in fracture resistance between designs, and the fracture pattern of most was involvement of pulp tissue and below the CEJ. Fracture resistance of the restored teeth was also not statistically different from the control. All control fractures were within the dentin and above the CEJ. Overlay restorations were therefore effective in strengthening damaged teeth and imparting fracture resistance equal to sound teeth, and axial wall heights and margin types did not influence this result.

## Introduction

Enamel and dentin are two main inorganic parts of a tooth, and each has unique characteristics that aid the other. The enamel is a strong and stiff substrate because it is 96% hydroxyapatite. The enamel sits on the dentin, which is more flexible but still resilient, containing 33% organic matter^1^. Both enamel and dentin undergo some degree of bending when chewing forces are loaded on them from several directions. The dentin provides the tooth as a whole with flexibility, helping protect the enamel from breaking, while the firmness of the enamel in turn protects the underlying vital parts, the dentin and pulp tissue, during chewing. This combined structure is uniquely effective. To date there are still no restorative materials that can replace this perfect combination of nature. Thus the original tooth structure should always be preserved as much as possible.

With the goal of limiting the amount of natural tooth structure removed, referred to as conservative treatment, dental bonded restorations have been developed to preserve as much of the intact tooth structure as is still in suitable condition to receive restorations^2^. A good dental adhesive system and resin cement are together the keys to bonding a ceramic or metal restoration to the natural tooth effectively. Bondable restorative materials can replace damaged tooth structure without resorting to the tooth reduction required to create of a mechanical lock, for which significant intact tooth structure is lost^3^.

An overlay is one kind of conservative treatment known as a partial coverage restoration, and the purpose of an overlay is to cover the whole occlusal surface of a tooth^4^. In cases of involuntary clenching or bruxism, tooth wear commonly occurs on the occlusal surface, leading to loss of the occlusal anatomy and reduced tooth height, and necessitating tooth rehabilitation^5^. Tooth preparation for a full coverage crown removes the intact tooth structure unnecessary for retaining the restoration^3^. An overlay is an alternative treatment with preparation of only part of the tooth for total coverage of all cusps. Bondable restorative materials, usually dental ceramics and resin-base materials are recommended for this kind of restoration along with a dental adhesive system and resin cement^5^. However, at present there are few clear recommendations for conservative tooth preparation before a ceramic overlay, with the desire to conserve as much of the tooth structure as possible in necessary balance with the desire for the restoration to have high fracture resistance to chewing forces.

The purpose of this study is to investigate fracture resistance of different axial wall heights and margin preparation designs for adhesive ceramic overlays. The null hypothesis is that different designs of overlay preparation have no effect on the fracture resistance of bondable glass ceramic restorations.

## Materials and methods

This study was approved by the Naresuan University Ethics Committee (Approval No. P10139/64). All methods were carried out in accordance with relevant guidelines and regulations. The written informed consent was waived for this retrospective analysis. The waiver of written informed consent for this retrospective analysis was approved by the Naresuan University Network of Research Ethics Committee.

### Sample preparation

The necessary number of samples was calculated with G*Power software set at power 0.8. Forty-eight sound human maxillary premolar teeth without cracks, restorations, or carious lesions (N = 48), which were previously extracted for orthodontic treatment, were selected for this study. The teeth were specifically selected for having similar measurements on the occlusal surface: 8.0–10.0 mm bucco-lingual distance, 7.0–9.0 mm mesio-distal distance, and 8.0–10.0 mm occluso-cervical distance. The teeth had been collected and stored in 0.1% thymol solution at room temperature, and all selected teeth were used within 3 months after extraction. All teeth were embedded in self-curing acrylic resin 3 mm below the cementoenamel junction.

**Figure 1:**
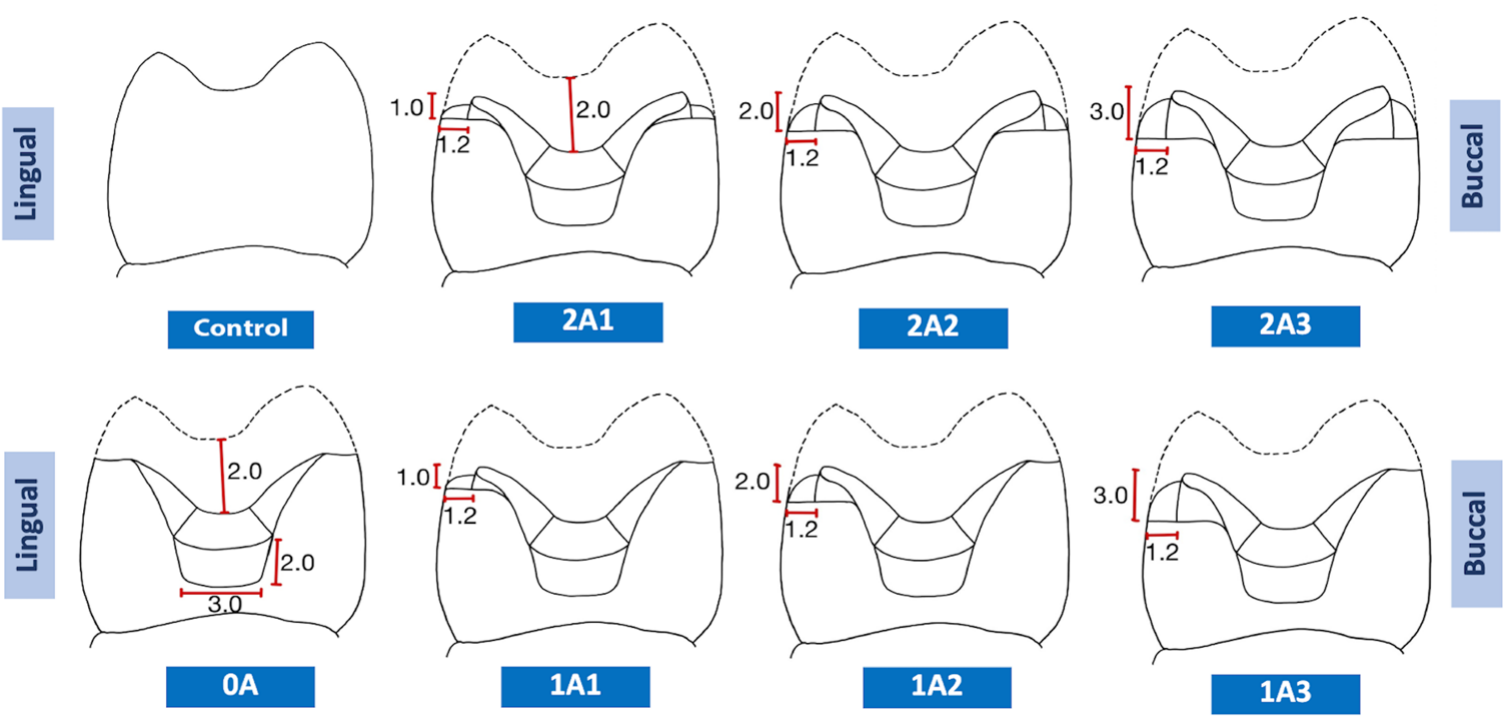
Illustrations of the various preparation designs in this study.

The teeth were then randomly divided into eight groups, shown in Table 1. Each group represents a specific combination of axial wall heights and margin preparation types. The control group had no tooth preparation. The various preparation designs are shown in Table 1 and Fig. 1.

**Table 1:** Characteristics of the various preparation designs in this study.

Groups	Height of axial walls		Types of margin preparation	
	Buccal	Lingual	Buccal	Lingual
Control	No preparation			
2A1	1 mm	1 mm	Shoulder	Shoulder
2A2	2 mm	2 mm	Shoulder	Shoulder
2A3	3 mm	3 mm	Shoulder	Shoulder
1A1	0 mm	1 mm	Contrabevel	Shoulder
1A2	0 mm	2 mm	Contrabevel	Shoulder
1A3	0 mm	3 mm	Contrabevel	Shoulder
0A	0 mm	0 mm	Contrabevel	Contrabevel

A high-speed handpiece with 10-degree taper diamond burs was used to prepare MOD cavities with a divergence of 10 degrees, a width of 3 mm at the pulpal wall, and a depth of 2 mm. The occlusal cavity was continuous with the proximal cavity with an angle of departure of 120 degrees and gingival margin width of 1.2 mm. The gingival walls of these proximal cavities were 2 mm below the pulpal floor.

All cusps were reduced 2 mm following the incline plane. The next step of the cavity preparation was to create the varied heights of the axial walls with a 1.2 mm shoulder margin. The heights of the buccal axial wall and lingual axial wall were various combinations of 1 mm, 2 mm, and 3 mm, as shown in Table 1. The groups with a shoulder margin on only the lingual wall (Groups 1A1, 1A2, and 1A3) had a contrabevel margin on the buccal wall. When there was no axial wall at all (Group 0A), there were contrabevel margins on both the buccal and lingual walls. All angles of the preparations were rounded with superfine diamond burs. There was no preparation and no restoration on the control group. They were sound teeth.

### Fabrication of the restorations

**Figure 2:**
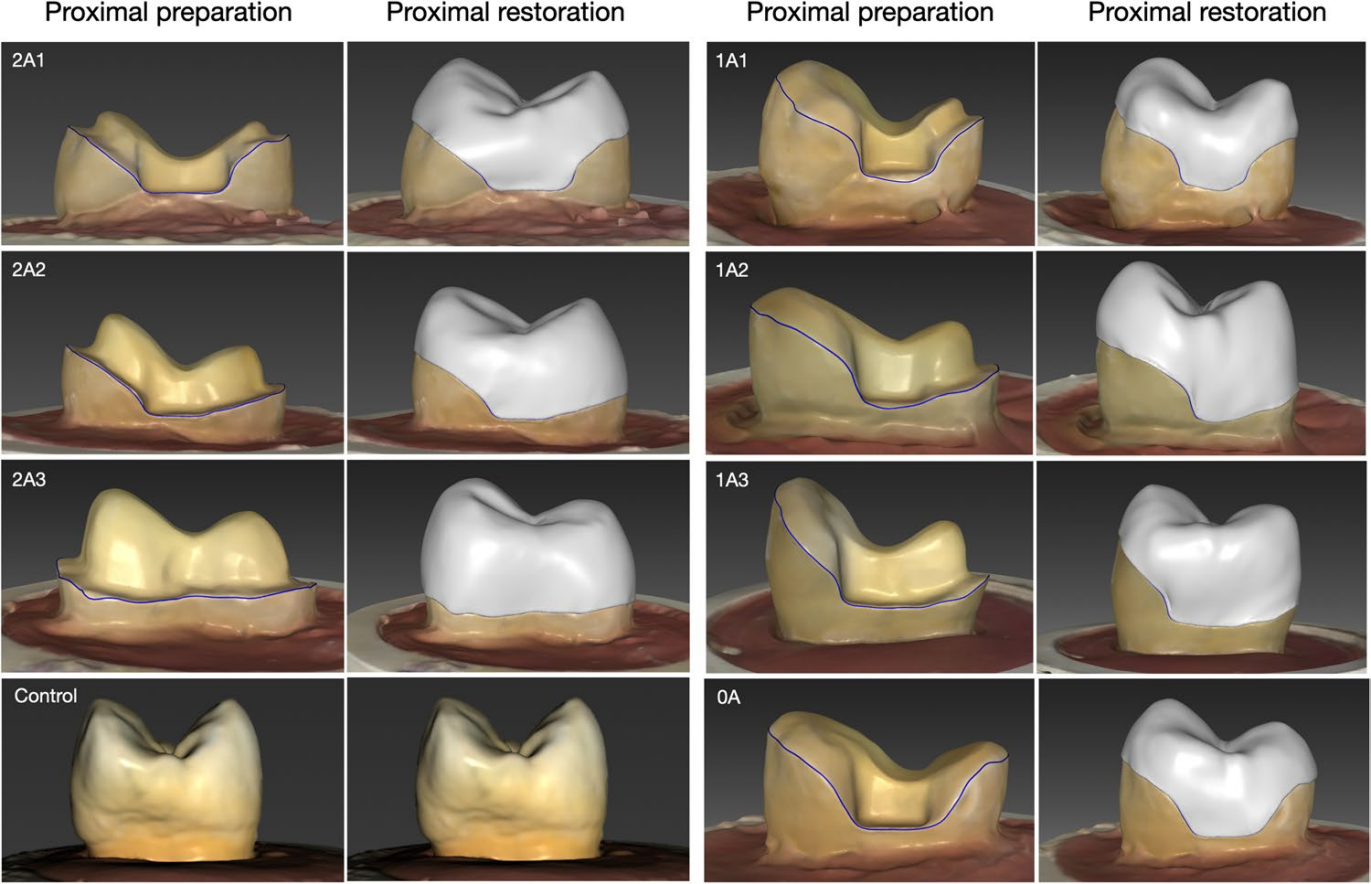
The tooth preparation and the overlay restoration design for each experiment group.

Before cavity preparation, all teeth were scanned with an intraoral scanner in the Biogeneric Copy mode (CEREC Primescan, Dentsply Sirona, Bensheim, Germany). After cavity preparation, the samples were scanned again, and then restorations were designed with Computer-Aided Design (CAD) software (CEREC SW 5.2, Dentsply Sirona, Bensheim, Germany), using the biogeneric copy from the initial scan to recreate the natural features of the original, unprepared tooth in the restorations. All overlay restorations were created from zirconia-reinforced lithium disilicate ceramic shade A2 (Celtra Duo^®^, Dentsply Sirona, Bensheim, Germany) in a milling unit (Primemill, Dentsply Sirona, Bensheim, Germany). After the milling process, heat treatment was performed using a sintering furnace (CEREC SpeedFire, Dentsply Sirona, Bensheim, Germany) at a temperature of 820 °C for 10 min and 45 s. The tooth preparation and the overlay restoration design for each experiment group are shown in Fig. 2.

### Cementing the restorations

Before cementation, all the zirconia-reinforced lithium disilicate ceramic restorations were examined under a stereomicroscope (SZX-ILLD200, Olympus, Tokyo, Japan) to ensure there were no cracks or surface flaws. The intaglio surfaces of the restorations were etched with 4.9% hydrofluoric acid (Porcelain Etch®, Ultradent, USA) for 20 s and then thoroughly rinsed with water for 30 s, followed by air-drying. The etched surfaces were then treated with Clearfil Ceramic Primer Plus for 20 s (Kuraray Noritake Dental, Inc., Tokyo, Japan) followed by air-drying.

The selective etch technique was used by applying 37% phosphoric acid to the enamel for 15 s. After that, the teeth were rinsed with water for 30 s, and excess water was removed by blow drying for 15 s. Tooth primer was applied and allowed to dry for 30 s, followed by application of resin cement (Panavia V5, Kuraray Noritake Dental Inc., Tokyo, Japan). The self-etch resin cement was used according to the manufacturer’s instructions and applied on the intaglio surface of the restorations, which were then seated with finger pressure. After tack curing with a light-curing unit (Demi^TM^Plus L.E.D., Kerr Corporation, USA) for 3 s on each surface, the excess cement was removed. The restoration cement was then cured for 20 s on each surface. Finally, the margins of the restorations were finished and polished with a ceramic polishing kit (Luster for silicate ceramics, Meisinger, USA). After cementation, all specimens were stored in water at 37 °C for 7 days.

### Investigation of fracture resistance

**Figure 3:**
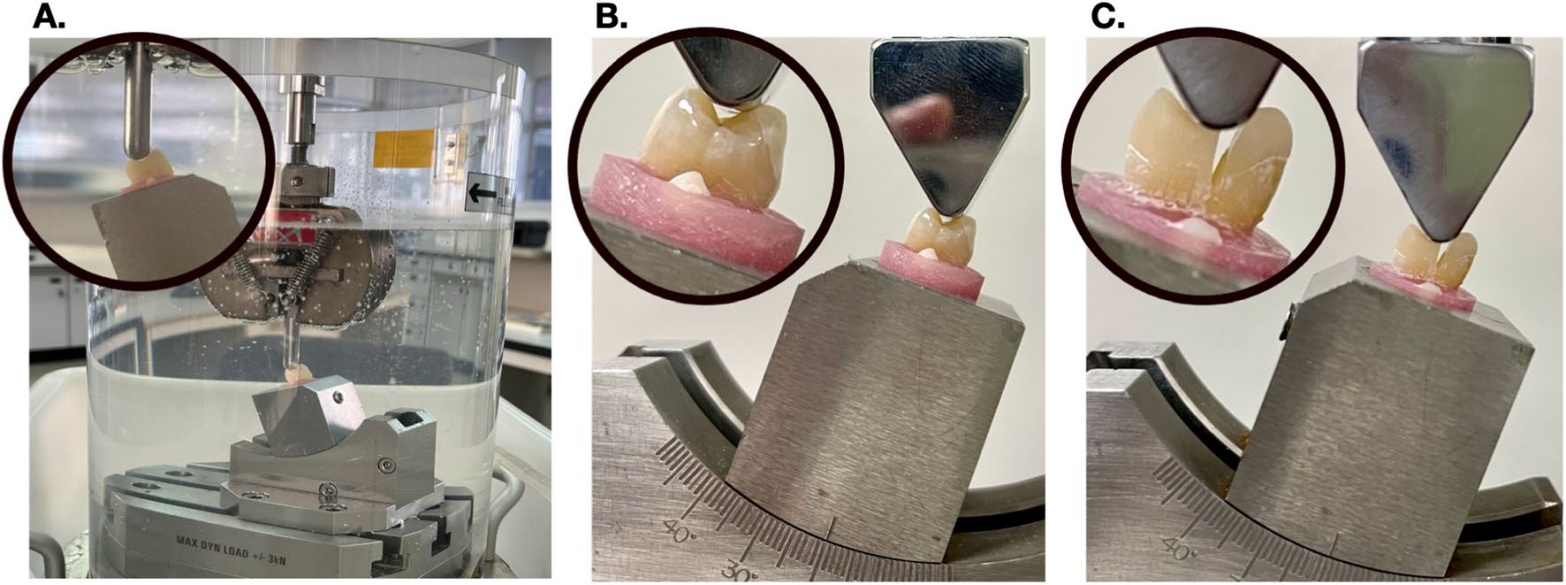
The specimens subjected to dynamic load in distilled water (**A**). The fracture resistance test on sound tooth (control) (**B**) and restored tooth (**C**).

All specimens were thermocycled in water (SDC20 HWB332R, Yamatake Honeywell, Japan) between 5 and 55 °C with 15 s dwelling time for 5000 cycles. This thermocycling represented approximately 6 months of in vivo functioning^6^. After thermal cycling, a fatigue simulation was performed in a universal testing machine (Instron Universal Tester; model 8872; Instron Inc., Canton, MA, USA) by subjecting the specimens to dynamic loading of 127.4 N with a metal sphere 6 mm in diameter at a frequency of 6 Hz^7^. During this fatigue simulation, the specimens were submerged in distilled water at 37 °C for 120,000 cycles (Fig. 3). A fracture resistance test (Instron Universal Tester; model 5965; Instron Inc., Canton, MA, USA) was then performed on the specimens by submitting them to a shear force on the buccal cusp. A blunt, wedge-shaped metal instrument was positioned at a 20° angle to the long axis of tooth.^3^ The wedge contacted both the lingual inclined plane of the buccal cusp and the buccal inclined plane of the lingual cusp. This wedge pressed down continuously on each specimen submerged under distilled water with a cross-head speed of 0.5 mm/min until fracture occurred (Fig. 3).

The fractured specimens were examined under a stereomicroscope (SZX-ILLD200; Olympus, Tokyo, Japan) to determine the fracture pattern both horizontally and vertically, following the method of Burke et al., somewhat modified^8^. The fractures were classified according to the following system, with each fracture assigned both a single “Fracture mode by involvement” and a single “Fracture mode by location”:

1. Fracture modes by involvement in restoration, dentin, or pulpal tissue
  Type I: Fracture within the restoration (or enamel in the case of the control).
  Type II: Fracture of the restoration and dentin.
  Type III: Fracture of the restoration and tooth structure reaching beyond the dentin and involving pulpal tissue.
2. Fracture modes by location above or below the CEJ
  Type I: Fracture above the CEJ.
  Type II: Fracture below the CEJ.

### Data analysis

Statistical software (SPSS 23.0, SPSS Inc., Chicago, IL, USA) was used to calculate the means and standard deviations of the fracture resistance in all groups. Data analysis showed that the fracture resistances were not normally distributed according to the Shapiro–Wilk test. The Kruskal–Wallis test was used to determine the significant difference in fracture resistance among eight groups. The fracture patterns were evaluated and reported descriptively.

## Results

### The fracture resistance of the various overlay designs

**Figure 4:**
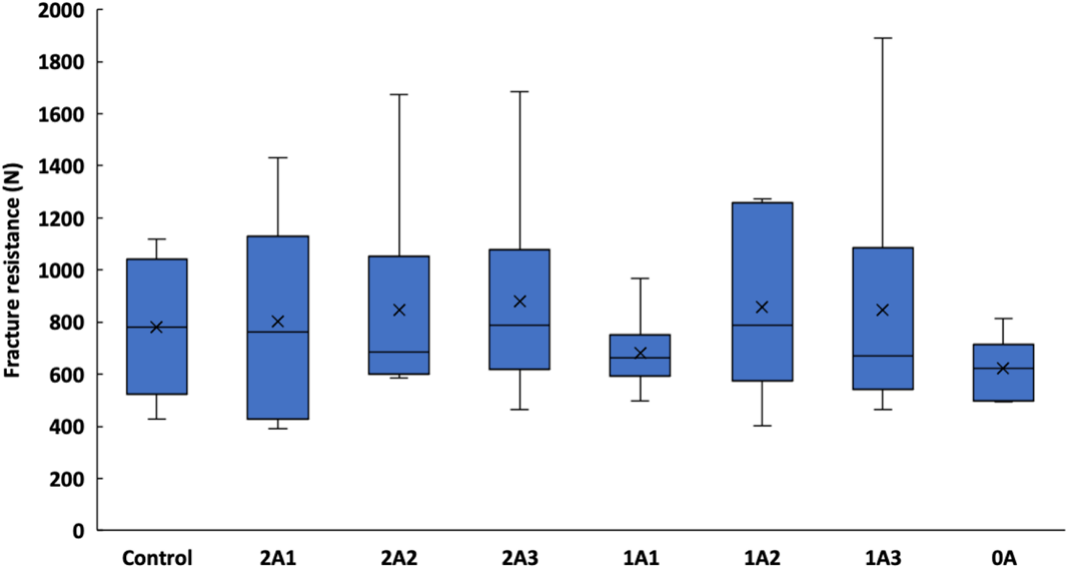
The fracture resistance of zirconia-reinforced lithium disilicate ceramic overlays restored on various cavity designs, compared with unaltered teeth (Control).

The zirconia-reinforced lithium disilicate ceramic overlays varied in the height of the axial walls and in the margin designs, as designed during cavity preparation. Teeth without any preparation were used as the control group. The highest median value of fracture resistance (787.523 N) was found in Group 2A3, which had two 3-mm high axial walls and two shoulder margins, and this was also higher than the control (780.112 N). The lowest median value of fracture resistance (624.031 N) was found in Group 0A, which had no axial walls on the buccal and lingual surfaces. However, there was no statistical difference between the groups (*p* = 0.8995) (Fig. 4).

### Analysis of the fracture mode

**Figure 5:**
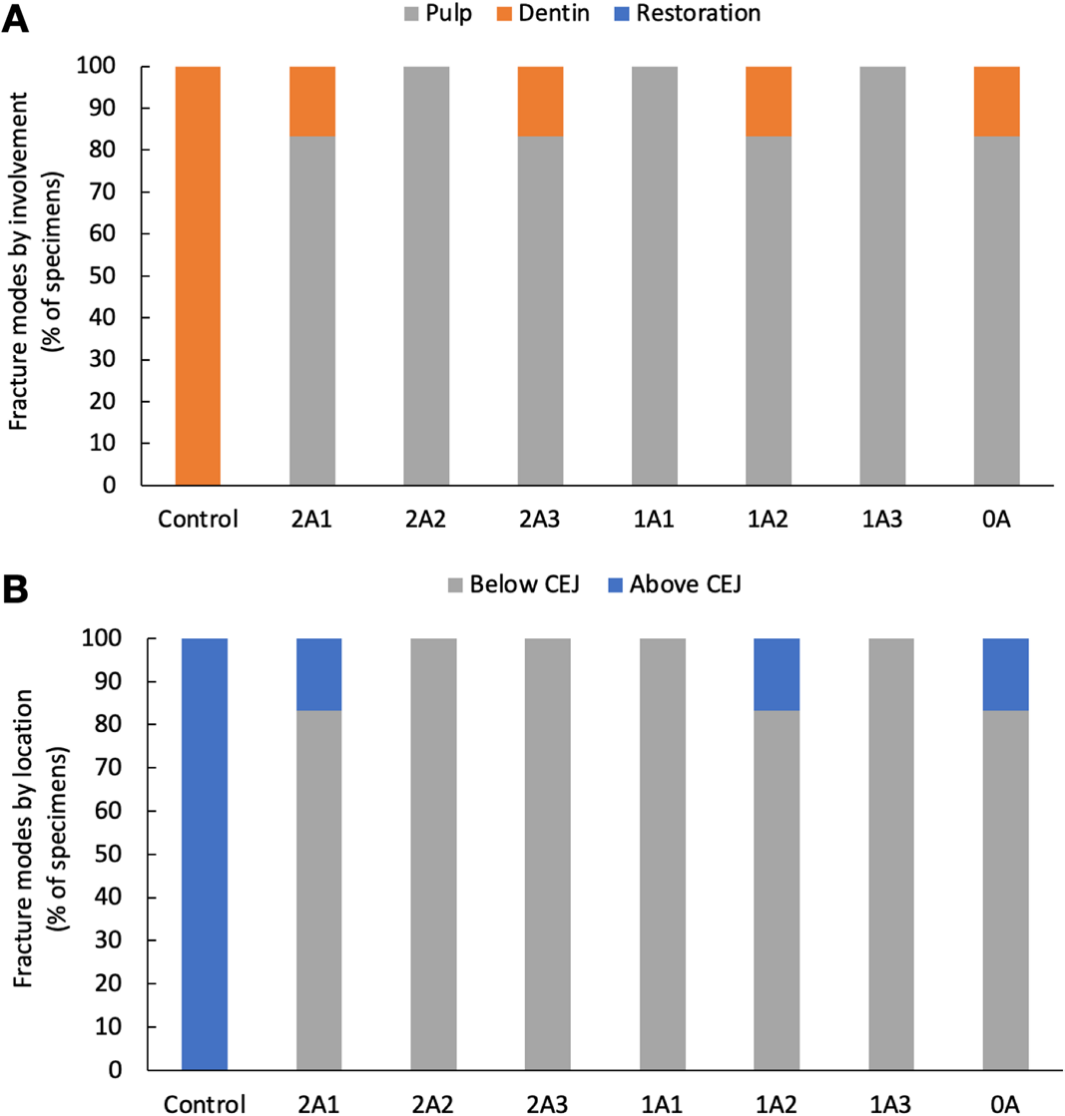
The fracture mode of the control group and the variously designed overlay groups according to (**A**) involvement and (**B**) location above or below the CEJ.

**Figure 6:**
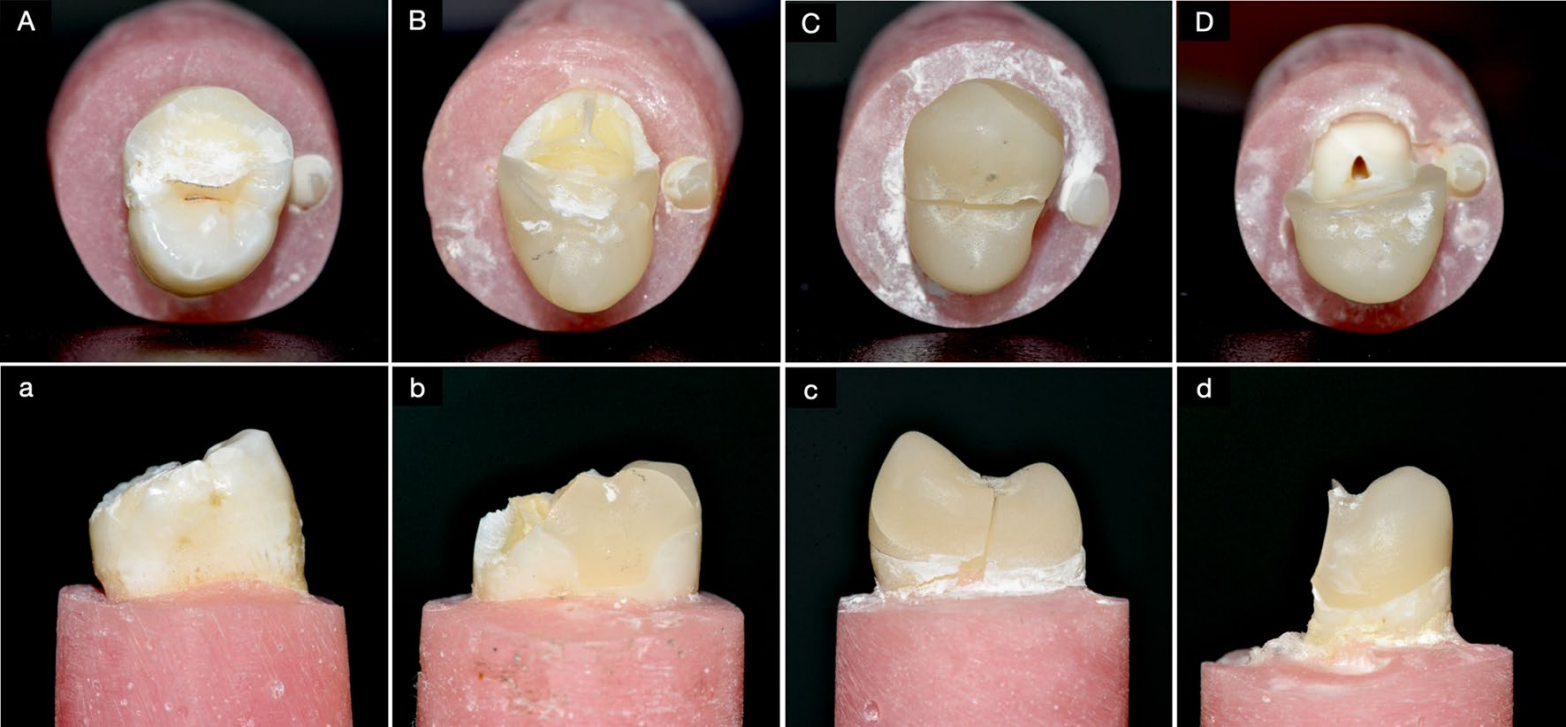
The characteristics of the observed fractures are shown in occlusal view (uppercase letters) and proximal view (lowercase letters). In the control group, the fracture was always within the dentin (**A**) and it was always above the CEJ (**a**). In the overlay-restored groups, four of the forty-two specimens involved only the restoration and dentin (**B**) and three of these unusual specimens were above the CEJ (**b**). All of the other overlay-restored specimens fractures involved pulp tissue (**C** and **D**) below the CEJ (**c** and **d**). Roughly half of those specimens fractured across the middle of the tooth (**C** and **c**), and in the others a large portion of the tooth fell off (**D** and **d**).

After the specimens underwent thermocycling and mechanical fatigue equivalent to 6 months of normal use, their fracture resistance was tested, and the characteristics of the fractured specimens were examined and analyzed (Figs. 5 and 6). Every specimen in the control group broke within the dentin layer, not reaching the dental pulp, and the location of the fracture line was above the CEJ. Among all the restored groups, almost all samples in each group had fractures involving the pulp tissue and below the CEJ. Only one specimen each in 2A1, 2A3, 1A2, and 0A had a fracture involving only the dentin. Those unusual specimens also broke above the CEJ, except for that single 2A3, which broke below the CEJ.

## Discussion

This study found no statistical difference in fracture resistance between the different designs of ceramic overlay restorations tested, and their fracture resistance was also not statistically different from that of the sound teeth in the control. Therefore the overlay restorations using the adhesive system were able to strengthen the defective teeth to a level of fracture resistance comparable to the sound teeth, while the type of margin and the length of the axial wall did not influence the final fracture resistance. Developmental advances in dental adhesive systems and in restorative materials have resulted in restorations being bondable to the natural tooth structure with no need for mechanical retention. A similar result was found in the study in 2018 by Hoopes et al. There was no statistic difference in the fracture resistance among 2-, 3-, and 4-mm height of axial walls in which adhesive ceramics were restored on the human molar teeth with shoulder margin.^9^. The adhesive technology can compensate for the reduced axial wall height in the case of conventional techniques^9^. Even though there was no difference in fracture resistance between different designs in this study, the 2A3 group (3-mm high buccal and lingual walls) had the highest fracture resistance while the A0 group (no axial walls on buccal and lingual surfaces) had the lowest fracture resistance. This result was similar to the study in 2018 by Hoopes W. et al., which found that the 1-mm high axial wall had significantly lower fracture resistance compared to 2-, 3-, and 4-mm high axial wall^9^. Therefore an axial wall height of at least 2-mm is recommended when the remaining tooth structure requires strengthening, such as with a cracked tooth or with a root canal treated tooth.

The bonding of a restoration has more influence on its clinical success than the strength of the restoration materials used^10,11^. All restoration samples were human teeth restored with ceramic overlays and then placed under a stress test to determine the fracture resistance. On observation of the fracture patterns in this study, the dominant pattern of the restoration samples was located between the buccal and lingual cusps, which involved the pulpal tissue and location below the CEJ, regardless of which design was used. The fracture pattern of the sound teeth was in all cases limited to the dentin of the buccal cusp and always above the CEJ. Even though the fracture resistance of the restored teeth and the sound teeth were not statistically different, they clearly had different fracture patterns. Their respective patterns resulted in the fractured overlay specimens being rendered clinically unrestorable while the fractured control specimens were still restorable. This difference demonstrates and highlights the importance of preserving natural tooth structure. The more natural tooth structure that remains, the better the long-term prognosis for that tooth^12^. In clinically restorative treatment, adhesive restoration can strengthen the remaining tooth, however the preservation of natural tooth structure is key to the longevity of the tooth.

It is recommended that compromised teeth such as root canal treated teeth or cracked teeth be restored with full cuspal coverage restorations^13–15^, of which the overlay restoration is one type. The full cuspal coverage restorations can disperse occlusal forces over the entire tooth surface to minimize the local bearing stress^16^. In this study the shear loading was applied to the samples with continuous loading until fracture. This is different from actual daily function, which normally has a resting period. The fracture of ceramic can occur suddenly resulting from the cumulatively extended period of low loading^17,18^. Therefore the continuous laboratory loading of the ceramic in this study was not different from the clinical function of the ceramic in regard to the fracture of the ceramic. The laboratory continuous loading seems to require a smaller period of time in observing the fracture resistance of the ceramic.

The material used for the overlay restorations in this study was zirconia-reinforced lithium disilicate ceramic, which is lithium disilicate ceramic with 10% zirconium dioxide. Lithium disilicate ceramic is a glass-based ceramic which, in conjunction with intermediate layers of resin cement and dental adhesive, has micromechanical retention to tooth structure^19^. Hydrofluoric acid is applied to the surface of the lithium disilicate ceramic, which causes the glass phase to dissolve and the tiny lithium disilicate crystals to be exposed. This creates a rough surface^19^. Silane is then applied onto this rough surface of the ceramic, where it will form covalent siloxane bonds between the hydroxyl groups of the ceramic and the C=C bonds in the resin cement^20^. Together, this series of steps results in microscopic mechanical retention between the lithium disilicate ceramic and the tooth structure^19^.

The mechanical properties of zirconia-reinforced lithium disilicate ceramic make it highly suited to restorative work. The pre-crystalized lithium disilicate glass ceramic block (which is blue) is composed of platelet-shaped lithium metasilicate crystals (Li_2_SiO_3_) (40% w/w, size = 0.2–1 μm) and needle-shaped lithium disilicate crystals (Li_2_Si_2_O_5_) (0.8 μm × 5 μm), the latter functioning as nuclei in the glass matrix. In the crystallization process, heat turns the metasilicate crystals into the disilicate form, for a total lithium disilicate crystal content of approximately 70%. The crystallization and nucleation of the lithium disilicate crystals increase the material’s flexural strength (from 130 to 360 ± 60 MPa) and fracture toughness (from 0.8–1.2 to 2–2.5 MPa m^1/2^)^21^. These higher mechanical properties result from the tightly interlocked disilicate crystals, which hinder crack propagation within the material^22^. Zirconia-reinforced lithium disilicate ceramic is an improvement on the earlier lithium disilicate ceramic. It contains 10% (w/w) tetragonal zirconium oxide (ZrO_2_) crystals (size 80–200 nm), long lithium metasilicate, and round granule lithium orthophosphate (Li_3_PO_4_)^23–26^. The zirconia particles reinforce the ceramic structure by functioning as a crack interruption^23,26^, thereby strengthening the material as a whole. After the crystallization process, there are lithium disilicate, diphosphorus pentoxide (P_2_O_5_), and lithium-zirconium silicate glass ceramics (Li_2_O–ZrO_2_–SiO_2_)^23^. The P_2_O_5_ functions as a nucleation agent in the crystallization^19^. The Li_2_O–ZrO_2_–SiO_2_ has fine grain structure which is 4–8 times smaller than the lithium disilicate crystals^23^. This ultra-fine microstructure with high glass content makes zirconia-reinforced lithium disilicate ceramic a translucent material with high flexural strength and a visually harmonious crystalline structure^21,26^. The fractural resistance and fracture toughness of zirconia-reinforced lithium disilicate ceramic have been previously determined to exceed 560 N^27–30^ and 152 MPa m^1/2^, respectively^24,25,28,31^.

The amount of tooth structure that needs to be removed depends on the type of materials used. Lithium disilicate ceramic and tooth enamel have a similar modulus of elasticity, approximately 80 GPa. This similarity is very advantageous when lithium disilicate ceramic bonds to tooth enamel. There is less tension stress during chewing, and that could reduce the risk of ceramic fracture^32,33^. The fracture risk of a thin 1 mm layer of lithium disilicate ceramic cemented onto enamel has been previously found to be lower than that of thicker restorations (1.5–2 mm as recommended) cemented onto dentin due to lower mechanical complication^34–36^. In a study monitoring normal clinical use of thin lithium disilicate restorations for 32 months, no fractures occurred in these restorations, even though in some cases their thickness was 1.1 mm at the dental cusp and 0.7 mm at the fossa^33^.

The full occlusal coverage of overlay restorations has been proven to enhance the fracture resistance of root canal-treated teeth^37–39^, which have decreased strength and are more prone to fracture^40,41^. An overlay is often a good conservative option for a root canal-treated tooth missing a large amount of original tooth structure, because these teeth usually have reduced cuspal flexure and decreased fracture resistance in the remaining tooth structure^42,43^. Overlay restorations have also been shown to distribute biting forces evenly across the tooth, and they have good cavity configuration (C factor) that results in low polymerization shrinkage stresses at the adhesive interface^39^. Another good feature is that overlays require only occlusal reduction, not removal of all surrounding tooth surfaces as required in the case of a full crown. When a tooth has large occlusal wear facets, such as those caused by erosion or attrition, and a substantial amount of missing coronal tooth structure, the treatment aim is to replace the lost tooth structure. Patients in this situation benefit from using an overlay to reconstruct the occlusal surface and preserve the surrounding tooth structure^44^. The more natural structure that can be preserved, the greater the longevity of the tooth will be. Since the nature of this restoration involves reconstructing tooth surface that has been lost, the restorations are in cavities which cannot be used for mechanical retention, so bondable restorations must be used. In this situation, glass ceramic is recommended as a restoration material because of its high shear bond strength to enamel and dentin, 25–37 MPa^45^ and 12–45 MPa^46,47^, respectively.

The clinical survival rate for lithium disilicate ceramic restorations after a year of use was previously found to be 85%, with fracture of the restoration being the main cause of failure^48^. Although the current study found that the height of the axial walls (0, 1, 2 or 3 mm) did not cause a statistically significant difference in the fracture resistance of the restorations, higher axial walls did facilitate correct insertion of the restorations, preventing dislocation during the process. Therefore if the axial walls are short, particular care is necessary when placing the restoration on the abutment. Another independent variable in this study was the type of margin. A contrabevel margin preserves much more of the natural tooth structure than a shoulder margin. Previous studies have recommended placing a shoulder margin so that it wraps around the functional cusp(s), since functional cusps are more prone to fracture^49,50^, particularly the lingual cusps^51^. When a lingual cusp fractures, total fractures were previously found to occur 3.2 times more frequently than partial fractures, and subgingival fractures were found to occur 3.62 times more frequently than supragingival fractures^51^. However, the current study found that regardless whether lingual cusps were wrapped with shoulder margins, the fracture resistances did not differ.

## Conclusion

This study found that the height of axial walls and the type of margin used in bondable ceramic overlays had no significant influence on the fracture resistance of the restorations.

## Data Availability

All data generated or analysed during this study are available on request from the corresponding author, upon reasonable request.
